# The Influence of Immediately Loaded Basal Implant Treatment on Patient Satisfaction

**DOI:** 10.1155/2020/6590202

**Published:** 2020-04-14

**Authors:** Fadia Awadalkreem, Nadia Khalifa, Asim Satti, Ahmed Mohamed Suleiman

**Affiliations:** ^1^Department of Oral Rehabilitation, Prosthodontic Division, University of Khartoum, Faculty of Dentistry, Khartoum, Khartoum, Sudan; ^2^Department of Preventive and Restorative Dentistry, University of Sharjah, Faculty of Dental Medicine, Sharjah, UAE; ^3^Department of Computing and Research, Federal Ministry of Health, Khartoum Teaching Dental Hospital, Khartoum, Khartoum, Sudan; ^4^Department of Oral and Maxillofacial Surgery, University of Khartoum, Faculty of Dentistry, Khartoum, Khartoum, Sudan

## Abstract

**Background:**

Improving patient satisfaction and quality of life is of great importance when considering the different prosthetic treatment options for patients with severely resorbed residual alveolar ridges. We aimed to evaluate and compare patients' satisfaction when changing from fixed, removable, and/or conventional implant prostheses to basal implant-supported prostheses.

**Methods:**

Sixty patients with a history of fixed, removable, and/or conventional implant prostheses who received basal implant-supported prostheses (BCS®, IHDE Implant System) were included in this study. Direct interviews were conducted using a four-section questionnaire that covered sociodemographic data, clinical examination, information on previous prostheses, and new implant information. The obtained data were statistically analysed using a Wilcoxon signed-rank test and chi-squared test.

**Results:**

Patients were predominantly female, partially edentulous, and aged between 40 and 59 years. Patients' general satisfaction with basal implants was very high (7.7 out of 8). Patients' satisfaction with comfort, mastication, speech, and aesthetics significantly improved with the new basal implants. Males aged between 40 and 59 years and patients who had previously used both fixed and removable prostheses were generally the most satisfied. Although some patients had complaints, they still had high satisfaction and would choose the same treatment modality again.

**Conclusions:**

Basal implant-supported prostheses have a positive impact on oral health and highly increase patients' satisfaction.

## 1. Introduction

The ultimate goal of dental and orofacial treatment is not only to treat oral disease but also to improve patients' quality of life [1]. Tooth decay, periodontal disease, trauma, tumour resection, and orthognathic treatment are the most common causes of tooth loss [2] resulting in aesthetic, functional, psychological, and social implications [2–4] that reduce patients' quality of life [5, 6].

Many prosthetic options have been made available for replacing missing teeth, including fixed, removable (acrylic and metallic dentures), and implant-supported prostheses [7, 8]. The choice between the different options depends on many factors such as the patient's age, gender, medical condition, occupation, socioeconomic status, number and position of missing teeth, condition of the remaining teeth, opposing dentition, quality and quantity of residual bone, dentist and technician expertise, and patient preference [9].

Fixed prostheses and removable dentures have been the traditional methods for replacing missing teeth [7, 8]. However, in cases of severe ridge resorption, these methods have many drawbacks, such as loss of retention, instability, difficulty in mastication, speech problems, and patient discomfort—all issues that negatively impact patient satisfaction [10–12].

With recent advances in dentistry, implants are now considered the gold standard treatment for replacing missing teeth. Many implant systems have been developed and distributed in the dental market, one of which is the basal implant [12–22]. In this system, the implant is anchored to the basal/cortical bone [12–22] which is useful in cases of severe alveolar ridge resorption, when bone grafting is prohibited due to the patient's general medical condition and when a more conservative treatment with lower cost is needed [12–14, 16, 20–22]. The BCS® implant is a special type of basal implant, consisting of one piece that is inserted through a crestal approach, just like the other endo-osseous implants and then anchored deeply inside the basal bone through its horizontal plates [12–14, 19]. Lazarov [13] revealed in a prospective cohort study that the use of Strategic Implant® prosthesis (BECES/BCS, KOS, KOS Plus, and BOI) is a safe and efficient procedure with a high success rate and without peri-implantitis. He followed up 1019 BECES/BCS cases for more than 48 and up to 57 months and reported a cumulative survival rate of 97.5%.

Several studies [8, 11, 23–31] have been conducted to evaluate patients' satisfaction with endo-osseous implant-supported prostheses using a number of parameters including mastication, aesthetics, speech, comfort, and overall satisfaction, while other studies [32–40] have used quality of life questionnaires such as the Oral Heath Impact Profile and the Geriatric Oral Health Assessment Index to evaluate patient satisfaction and improvement in oral-health-related quality of life.

Although the use of basal implant-supported prosthesis has been documented as an alternative treatment for patients with severe ridge resorption [12–22], there is a paucity of knowledge on how this treatment affects patients' satisfaction and quality of life compared with their previous prosthetic treatment. To our knowledge, this is the first study to consider the evaluation of patient satisfaction following fixed immediately loaded basal implant-supported prosthesis. Therefore, this study aimed to evaluate and compare patients' satisfaction when changing from fixed, removable, and/or conventional implant prostheses to basal implant-supported prostheses.

## 2. Materials and Methods

### 2.1. Patient Selection and Informed Consent

The study was approved by the ethical committee of Khartoum Dental Teaching Hospital (Khartoum, Sudan) and the Sudanese Ministry of Health, State Khartoum, number: WK/OS/AETEA/44/1. The study was undertaken with the understanding and written consent of each participant and in accordance with the Declaration of Helsinki.

After approval, all the patients planning to receive BCS® basal implants (Dr. Ihde Dental AG, Gommiswald, Switzerland) at the Implant Department at Khartoum Dental Teaching Hospital between December 2015 and December 2017 were screened using the following criteria and were asked to enrolled in the study: (1) insufficient residual bone volume preventing the use of conventional implant unless preceded with a bone grafting procedure that was precluded due to patient general health, patient request for more conservative treatment, and/or financial circumstances; (2) history of wearing fixed, removable, and/or conventional implant prosthesis; (3) patient's willingness to participate in the study after a full description of the study protocol and signing the informed consent form.

### 2.2. Sample Size

The sample size for the study was calculated with confidence level 95% using the following formula.

(1) $$\begin{array}{c} n=\frac{z^{2*}p*q}{d^{2}}, \end{array}$$

where *d* = desired margin of error 5%, *p* = prevalence, *q* = 1 − *p*, *z* = critical value of significance level, and *n* = sample size. *P* = 3% (the prevalence of population with prostheses in Sudan as reported with Khalifa et al.) [41]:

(2) $$\begin{array}{c} n=\frac{1.96\times 1.96\times 0.97}{0.05\times 0.05}=44.72. \end{array}$$

However, to increase the power of the study, the sample size rounded to 60 Patients.

### 2.3. Surgical and Prosthetic Procedure

**Figure 1:**
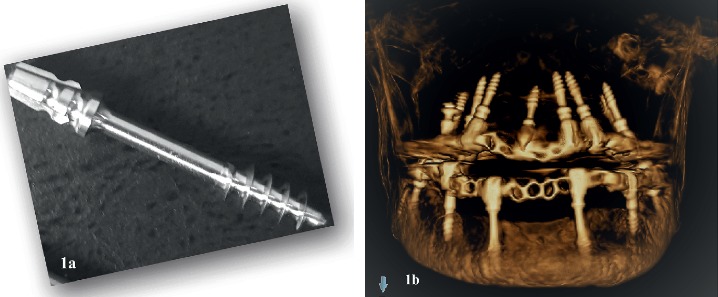
(a) BCS® basal implant design. (b) A three-dimensional cone-beam computed tomography image shows the anchorage of the BCS® implants within the basal bone in patients presented with a severely resorbed alveolar ridge.

All the patients were treated by the same maxillofacial surgeon and prosthodontist. Implant osteotomy was performed under infiltration local anaesthesia using the flapless technique. Three to ten BCS® basal implants (3.5 or 4.5 mm width × 14, 17, 20, 23, 26, and 29 mm length) were inserted in each jaw using the conventional protocol (Figures 1(a) and 1(b)). Implant length and width were determined using panoramic and cone beam computed tomography (CT) views. The primary fixation torque was 35 Ncm for all the implants. Implants were splinted using a metal framework, over which an acrylic or porcelain veneer material were added according to the hard and soft tissue loss. Immediate functioning circular and/or segment bridges were constructed and cemented within 3 days of insertion. Patients were provided with oral hygiene instructions, and follow-up visits were planned at 1 week and 1, 3, 6, and 12 months thereafter. At each follow-up visit, both clinical and radiographical examinations were conducted. Complications were reported and dealt with.

### 2.4. Questionnaire Design

Direct interviews were conducted using a questionnaire published by Zitzmann and Marinello [26] with some modifications. Our questionnaire consisted of four sections. Section A contained seven sociodemographic items: patient's name, code, age, gender, occupation, residence, and telephone number. Section B comprised the clinical examination of the patient (i.e., dental status chart). Section C contained previous prosthesis data: type of previous restoration, duration of prosthesis, evaluation of previous prosthesis (i.e., satisfaction with comfort, mastication, appearance, and speech), reasons for change, how the patient found out about the new implant system, and the patient's expectations for the new system. Section D contained basal implant data: evaluation of basal implant prosthesis (i.e., satisfaction with comfort, mastication, appearance, and speech), patient's complaints, dentist visits required after treatment, and probability of choosing this type of treatment again. Sections A, B, and C were completed before the implant treatment, while section D was completed after 1 year of prosthesis's functioning except the patient's complaint data, which were addressed in the first follow-up visit (one week after implant insertion).

### 2.5. Patient Satisfaction Measurement

Participants rated their level of satisfaction regarding comfort, speech, appearance, and mastication as excellent (2), average (1), or poor (0). The overall satisfaction was the sum of the patient's comfort, speech, appearance, and mastication scores, calculated for the previous prosthesis and the new basal implant; therefore, it ranged from 0 to 8.

### 2.6. Reliability and Validity of the Questionnaire

A pilot study was performed before the start of the study to investigate the internal consistency and the test-retest reliability of the questionnaire using the Cronbach Alpha test and the intraclass correlation coefficients (ICC), respectively [42]. The questionnaire was administered to 10 patients twice with two weeks' elapse interval. The Cronbach *α* was used to measure the consistency between the different questions and resulting in 0.755. On the other hand, intraclass correlation coefficients was calculated using scores from the repeated administration of the questionnaire resulting in 0.928.

### 2.7. Data Analysis

Data were collected, tabulated, and statistically analysed using IBM SPSS version 22. A *p* value < 0.05 was considered statistically significant. Wilcoxon signed-rank and chi-squared tests were used to analyse the data.

## 3. Results

### 3.1. Participants' Characteristics

After considering the inclusion criteria, a total of 60 patients were enrolled in the study, 37 (61.7%) of whom were female and 23 (38.3%) male. The age of the patients ranged from 20 to 73 years. Patients were categorised into three age groups, and the largest group was 40–59 years (34, 56.7%). Clinical examination revealed that half of the patients (51.7%) were partially edentulous (Table 1).

### 3.2. Participants' Knowledge of Basal Implants

Regarding how the patients had heard about basal implants, 90% had been referred to the implant department by other dentists, 11.7% had heard about implant treatments on the television, 3.3% were advised about implants by their friends, and 3.3% had read about implant treatments in newspapers and on the Internet (Table 2).

### 3.3. Participants' Expectations

Regarding their expectations about implant treatment, nearly all patients (98.3%) expected a fixed treatment modality, 49% expected to improve their mastication, 39% expected to improve their aesthetics, and 50% expected better retention of their prosthesis (Table 2).

### 3.4. Types of Previous Prosthesis

All patients had a history of tooth replacement: 35 (58.3%) had removable prostheses, 19 (31.7%) had fixed prostheses, 4 (6.7%) had had both fixed and removable prostheses, and 2 (3.3%) had conventional implant-supported prostheses (Table 3).

### 3.5. Reasons for Prosthesis Change

As for the reasons for changing their previous prosthesis, the main reasons for changing fixed prosthesis were caries/fracture of the abutment (65.2%) and poor retention (39.1%), while the main reasons for changing removable prosthesis were poor retention (56.4%) and patient discomfort (33.3%). Most patients mentioned more than one reason (Table 3).

### 3.6. Patient Satisfaction

**Figure 2:**
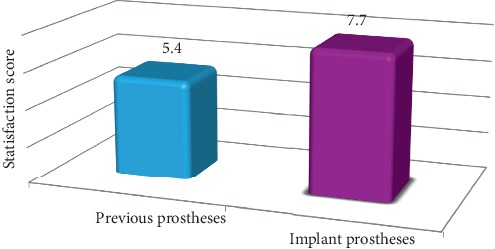
Participants' overall satisfaction with previous prostheses and current basal implant.

The Wilcoxon signed-rank test showed a statically significant difference between the mean scores of patients' overall satisfaction with the previous prosthesis (5.4 ± 1.7) and the basal implant (7.7 ± 0.7) (*p*=0.0001^*∗*^) (Figure 2, Table 4). The chi-squared test showed a statistically significant difference in patients' satisfaction with comfort, mastication, speech, and aesthetics when comparing the previous prosthesis with the basal implant (Table 4).

More than half of the patients (55%) evaluated their satisfaction with comfort with the previous prosthesis as average, whereas 96.7% rated it as excellent with the new implant (*p*=0.0001). Most patients (93.3%) assessed their satisfaction with mastication as excellent after the implant treatment, whereas 43.3% rated it as average with the previous prosthesis (*p*=0.0001). About half of the patients (56.7%) evaluated their satisfaction with the aesthetics of their previous prosthesis as excellent, which increased to 88.3% with the basal implant (*p*=0.0001). A total of 76.7% of the patients rated their speech with their previous prosthesis as excellent, which increased to 93.3% with the new implant (*p*=0.034) (Table 5).

### 3.7. Participants' Complaints

None of the patients needed or presented for an emergency visit after the implant treatment, although some presented at the follow-up visits with treatable complaints that were dealt with (Table 6). The following complaints were included: amount of teeth shown (3.3%), problem in S sound phonation (3.3%), difficulty in maintaining oral hygiene instruction (1.7%), discomfort (1.7%), and spaces between the teeth (1.7%) (Table 6). However, during their scheduled follow-up visits, all patients insisted they would choose the same treatment modality again.

### 3.8. Relationship between Satisfactions of the Participants and their Age and Gender

The Wilcoxon signed-rank test showed a statistically significant difference between previous and current prosthesis satisfaction for both genders (*p*=0.001^*∗*^, *p*=0.001^*∗*^) and across all age groups (*p*=0.004^*∗*^, 0.001^*∗*^, 0.007^*∗*^), and patients aged 40–59 showed a higher improvement in satisfaction than the other age groups (Table 7).

## 4. Discussion

The main goal of oral rehabilitation is not only to replace missing teeth with a prosthesis that will last for life but also to improve patients' quality of life and satisfaction. The latter relies on many factors, such as function (mastication and speech), comfort, aesthetics, and self-esteem [4].

According to the existing literature [8, 11, 23–31], patient satisfaction is evaluated using both general and specific questions that focus on a particular aspect in order to avoid the false-positive responses associated with general questions. The questionnaire used in this study contained both general parameters (overall satisfaction) and specific parameters most commonly used in the previous studies to investigate patients' oral health satisfaction, i.e., comfort, appearance, mastication, and speech [8, 11, 23–31].

The rehabilitation of patients with severe ridge resorption using implant-supported prosthesis presents a huge challenge. The treatment plan involves a bone grafting procedure to improve the bone-implant foundation area, but this procedure may be limited by the age and medical condition of the patient, the extension of the edentulous space, cost efficiency, surgeon expertise, donor site morbidity, and patient preference. Basal implants have been prescribed as an alternative treatment for these patients with a high success rate, less severe complications, and lower cost and number of surgeries [12–14, 20, 21]. There is an increased need for clinical research to evaluate the patient satisfaction and quality of life in relation to this treatment modality as a major parameter indicating implant success.

Most patients enrolled in this study were female, in line with the previous studies [41, 43, 44] reporting that females are more prone to dental caries, which is one of the main causative factors of tooth loss. Additionally, females tend to visit dental clinics more often than males, increasing the possibility of tooth extraction and edentulism [43, 44].

Khalifa et al. [41] reported a low percentage of complete edentulism among the Sudanese population, as individuals seemed to have extracted only teeth that hurt. Moreover, the high cost of implant prostheses for completely edentulous patients combined with low economic status may limit those seeking implant treatment to partially edentulous patients [41].

In accordance with other studies conducted by Saha et al. [45], Annibali et al. [31], Pommer et al. [46, 47], and Kohli et al. [48, 49], most of our patients were referred by other dentists. This could be due to the limited information available about implants in developing countries; therefore, dentists are still the main source of information about implants, followed by friends and online media. Thus, it is necessary to increase patients' awareness about implant treatment including basal implants.

Patients' expectations are an important parameter that has a great impact on their satisfaction [8, 31, 50]. Similar to other studies [11, 50–52], our results showed that patients' main expectations of basal implant treatment included having a fixed treatment modality and improving their mastication, aesthetics, and retention relative to their previous prostheses. Many authors [8, 11, 12, 20, 50] reported that, in cases of severe ridge resorption, conventional removable prostheses may have some drawbacks that might adversely affect the patient satisfaction, such as denture instability (especially the mandibular denture), inefficient mastication, poor retention, and discomfort. These drawbacks increase in the case of severe ridge resorption. On the other hand, several techniques have been advanced in order to optimise the aesthetic and functional outcomes of the prosthetic rehabilitation of patients with severe alveolar ridge resorption including the bone graft procedure [12, 13, 18, 20], use of short implants [16], use of “all-on-4 concept” [13], and utilisation of remote basal bone areas for anchorage such as the cortical bone of the nasal floor and maxillary sinus, pterygoid plate of the sphenoid bone, zygomatic bone, inferior cortex of the mandible and buccal and lingual cortex of the mandible for basal implants [12, 13, 18, 20].

The main reasons given by our patients for changing from a fixed conventional prosthesis were caries and fracture of the abutment, which is similar to numerous previous studies [24, 53–55]. Goodacre et al. [53] noted that the most common complications associated with conventional fixed partial dentures were caries, need for endodontic treatment, loss of retention, aesthetics, periodontal disease, tooth fracture, and prosthesis/porcelain fracture. Pjetursson et al. [23] reported in a meta-analysis that the most frequent complications with fixed prostheses were of biological nature, such as caries and loss of pulp vitality. De Backer et al. [54] reported that the most common fixed prosthesis complications were irreversible ones such as caries, loss of retention, fracture of the framework, abutment fracture, and periodontal and apical problems. Younes et al. [55] found that the most frequent complications encountered with resin-bonded dental prostheses were debonding, caries, and periodontal breakdown.

Basal implants are a special type of implant integrated mainly in the strongest basal bone, providing a high degree of support, stability, and retention to patients with severe ridge resorption, something that cannot be achieved with a removable prosthesis. Basal implants also allow for immediate restoration, which decreases patients' discomfort and omits the need for transitional or temporary restoration. This treatment also minimises the cost and time required, offering a more conservative approach compared with bone grafting procedures [12–22]. All of these factors may have contributed to the high overall satisfaction rates obtained in this study. Despite the lack of knowledge regarding patient satisfaction and quality of life in relation to basal implants specifically, the results of this study are in line with other conventional endo-osseous implant results [23, 28, 31, 36–39] indicating that patients' quality of life significantly improved after treatment with implant-supported prostheses.

The strongest anchorage obtained with basal implants offers stable occlusal units leading to good chewing function [12, 13, 16, 18, 21] Most of the patients in our study reported a significant improvement in their satisfaction with mastication from average to excellent after basal implant treatment, a finding that matches the findings of S. Ihde and A. Ihde [12, 18] and Scortecci [15] and is in accordance with other studies on endo-osseous implant treatment showing improved mastication with implant-supported prostheses [56–60].

Since speech is usually affected by edentulism, improving patients' speech is one of the main purposes of replacing missing teeth [11]. According to the literature on conventional implants [23, 30, 40], implant-supported prostheses improve patients' speech because of their limited tissue coverage and minimal or no interference with the tongue and lips and the fact that they do not require palatal or rugae area coverage. Our study showed that patients' satisfaction with speech significantly improved with basal implants. However, two of the patients in the study complained about their phonation when pronouncing the letter S. The same complaint was reported in the studies of Goodacre et al. [53] and Heydecke et al. [30] who observed that a greater number of speech problems occurred when restoring the maxillary arch with conventional fixed implant-supported prosthesis compared with removable implant-supported prosthesis. This was attributed to air escaping through the space required for oral hygiene maintenance between the edentulous ridge and the fixed implant prosthesis.

There was a significant improvement in patients' satisfaction with aesthetics after basal implant treatment, which is in accordance with the findings of Emami et al. [40], Zitzmann and Marinello [26], Gurgel et al. [25], and Annibali et al. [31] concluding that implant treatment produced a significant improvement in patients' satisfaction with aesthetics, eating, degree of comfort, and phonetics, as well as general satisfaction.

Two patients in our study complained about the small size of the artificial teeth. In general, in implant prosthesis construction, the artificial teeth are smaller than natural teeth in order to decrease the occlusal table, minimise or avoid the cantilever effect, prevent offset forces, and increase the axial loading. Out findings matched the occlusal considerations discussed in the studies of Misch and Wang [61], Kim et al. [62], Yi et al. [63], and Abichandani et al. [64].

Easy cleaning and oral hygiene maintenance are essential for maintaining good peri-implant health. All patients in this study were able to maintain their oral hygiene habits except for one who experienced some difficulty. This matches the results of Annibali et al. [31] and Pjetursson et al. [23] but is in contrast with Yi et al. [63] who reported that it was more difficult to maintain oral hygiene after implant prosthesis.

## 5. Conclusion

Despite the limitation of the relatively small sample size in the present study, the high level of patient satisfaction obtained suggests that basal implant-supported prostheses (BCS®) in edentulous and partially edentulous patients have a positive impact on patient satisfaction and hence enhance their quality of life. There were marked improvements in patients' overall satisfaction and specific satisfaction with comfort, aesthetics, mastication, and speech. Further research needs to evaluate patient satisfaction and the oral health impact of basal implants using a larger sample size and a longer follow-up period.

## Figures and Tables

**Table 1:** Participants' characteristics including patients gender, age, and dentition of the patients.

Variable	Number of patients	Percentage (%)
*Gender*		
Male	23	38.3
Female	37	61.7
Age (years)		
20–39	16	26.7
40–59	34	56.7
60 and above	10	16.6
*Dentition*		
Upper/lower complete edentulous jaws	17	28.3
One complete and one partially edentulous jaw	12	20
Upper/lower partially edentulous jaws	31	51.7

**Table 2:** Participants' knowledge and expectations regarding basal implants.

	Frequency	Percentage (%)
*Source of knowledge*		
Referred from another dentist	54	90
Television	7	11.7
Friends	2	3.3
Newspaper and internet	2	3.3
*Patients' expectations about implant treatment*		
Fixed modality	59	98.3
Improved retention	50	83.3
Improved mastication	49	81.7
Improved aesthetics	39	65

**Table 3:** Participants' previous prosthesis type (fixed/removable/conventional implant) and reasons for changing to new basal implant.

	Frequency	Percentage (%)
*Types of previous prosthesis (% out of 60 patients)*		
Removable prosthesis	35	58.3
Fixed prosthesis	19	31.7
Fixed and removable prosthesis	4	6.7
Conventional implant-supported prosthesis	2	3.3
*Fixed prosthesis (% out of 23 patients)*		
Caries/fracture of abutment	15	65.2
Decementation/debonding	15	65.2
Inability to chew properly	4	17.4
Discomfort	4	17.4
Need for fixed prosthesis	1	4.3
*Removable prosthesis (% out of 39 patients)*		
Poor retention	22	56.4
Discomfort	13	33.3
Inability to chew properly	8	20.5
Caries/ fracture of abutment	8	20.5
Need for fixed prosthesis	5	12.8
Aesthetics	1	2.6

**Table 4:** Participants' overall satisfaction with previous prostheses and current basal implant.

Prostheses	Mean	SD	95% CI	95% CI	*p* value
			Lower bound	Upper bound	
Previous prosthesis	5.4	1.7	4.9	5.8	0.0001^*∗*^
Current prosthsesis	7.7	0.7	7.5	7.9	

SD: standard deviation. Wilcoxon signed-rank test ^*∗*^*pvalue is significant*.

**Table 5:** Comparison of patients' satisfaction with comfort, mastication, aesthetics, and speech with previous prosthesis and current basal implant.

	Satisfaction with previous prosthesis			Satisfaction with basal implant			*p* value
	Excellent (%)	Average (%)	Poor (%)	Excellent (%)	Average (%)	Poor (%)	
Comfort	13 (21.7)	33 (55)	14 (23.3)	58 (96.7)	2 (3.3)	0 (0)	0.0001^*∗*^
Mastication	20 (33.3)	26 (43.3)	14 (23.3)	56 (93.3)	4 (6.7)	0 (0)	0.0001^*∗*^
Aesthetics	34 (56.7)	23 (38.3)	3 (5)	53 (88.3)	7 (11.7)	0 (0)	0.0001^*∗*^
Speech	46 (76.7)	13 (21.7)	1 (1.7)	56 (93.3)	4 (6.7)	0 (0)	0.034^*∗*^

Wilcoxon signed-rank test ^*∗*^*p* value is significant.

**Table 6:** Participants' complaints after basal implant treatment and probability of choosing the same treatment again.

		Number of patients	Percent (%)

Patients' complaints	Teeth shown	2	3.3
	S sound	2	3.3
	Difficultly in maintaining OHI	1	1.7
	Discomfort	1	1.7
	Spaces between teeth	1	1.7

Would you choose the same treatment again	Yes	60	100
	No	—	0

**Table 7:** Comparison of patients' satisfaction with comfort, mastication, aesthetics, and speech with previous prosthesis and basal implant by gender and age group.

	Previous prostheses		Basal implant		*p* value
	Mean	SD	Mean	SD	
Male	5.3	1.4	7.8	0.4	0.001*∗*
Female	5.4	1.4	7.6	0.4	0.001*∗*
Age (years)					
20–39	6	1.8	7.7	0.5	0.004*∗*
40–59	5.1	1.7	7.9	0.4	0.001*∗*
60 and above	5.1	1.4	7.3	1.3	0.007*∗*

SD: standard deviation. Wilcoxon signed-rank test ^*∗*^*p* value is significant.

## Data Availability

The data used to support the findings of this study are available from the corresponding author upon request.

## Abbreviation

BCS®: Basal cortical screw implant.
